# Combustion Characteristics of Physically Mixed 40 nm Aluminum/Copper Oxide Nanothermites Using Laser Ignition

**DOI:** 10.3389/fchem.2018.00465

**Published:** 2018-10-09

**Authors:** Florin Saceleanu, Mahmoud Idir, Nabiha Chaumeix, John Z. Wen

**Affiliations:** ^1^Mechanical and Mechatronics Engineering, University of Waterloo, Waterloo, ON, Canada; ^2^Institut de Combustion, Aérothermique, Réactivité et Environnement, Centre National de la Recherche Scientifique, Orleans, France

**Keywords:** nanothermite reaction, nanoparticle, laser ignition, heterogeneous combustion, flame propagation

## Abstract

This paper reports on the ignition and flame propagation characteristics of aluminum/copper oxide (Al/CuO) nanothermite at different packing density, manufactured from 40 nm commercial Al and CuO nanopowders. A 3.5 W continuous wave laser was used to ignite the samples in argon at atmospheric pressure, and a high speed camera captured the flame propagation. The high speed images revealed that the fast laser heating creates significant material ablation, followed by heat transfer along the heated surface. The bulk ignition occurs near the edge of the top surface, followed by the self-sustained burning. Lightly pressed powders (90% porosity) ignited in ~0.1 ms and the burning front propagated at around 200 m/s, while the dense pellets (40–60% porosity) ignited in ~1 ms and the burning front propagated at around 10 m/s. These results indicate that the reaction mechanism changes from mass convection to heat diffusion with increasing the packing density. The ignition and burn speeds of these Al/CuO nanothermites at different equivalence ratios (ERs), along with SEM images of pre- and post-combustion, illustrate that the homogeneity of the mixture is a critical parameter for optimizing the performance. The Al rich mixtures show significantly lower ignition delays and higher burn speeds.

## Introduction

Nanothermites, or metastable intermolecular composites (MIC), are highly energetic solid state materials with tunable combustion properties. A typical example is the Aluminum/Copper Oxide (Al/CuO) nanothermite, which is a good candidate for applications that involve propellants and explosives due to its fast ignition and gas release characteristics. The overall reaction is given by

$$\begin{array}{l} 2Al_{\left(s\right)}+3CuO_{\left(s\right)}\to \;{Al_{2}O_{3}}_{\left(l\right)}+3Cu_{\left(l-g\right)} \end{array}$$

The heat release is 4,075 J/g and the adiabatic flame temperature is 2,843°K (Fischer and Grubelich, 1998). Aluminum and copper oxide are mixed at the nano scale in order to enhance the reaction rates. Detailed description of the methods of preparation and characterization of the Al based nanothermites and their applications can be found in literatures (Rossi, 2015; Lafontaine and Comet, 2016).

The fast oxidation of aluminum in a thermite reaction can occur in (1) condensed phase, where the oxygen ion is transported across the Al/CuO interface, and (2) gas phase, where the CuO first dissociates, and then O_2_ gas reacts with Al in a similar way that Al oxidizes/burns in an oxygen rich atmosphere. Once the Al/CuO is ignited, the combustion flame self-propagates via mass and heat transfer processes such as thermal conduction and mass convection. Constant volume cell experiments illustrated that the pressure signal leads the optical emission signal, since CuO can fully decompose at temperatures below the adiabatic flame temperature (Sullivan and Zachariah, 2010). Also, electrically heated nanothermite coatings showed that CuO decomposes prior to ignition but only at heating rates higher than 2,000°K/s (Williams et al., 2013). Moreover, lightly packed Al/CuO powders was shown to have three modes of combustion with increasing the surrounding inert gas pressure, where the combustion velocity decreased from 1,000 to 2 m/s (Weismiller et al., 2009). Convection was shown to be dominant at low (near atmospheric) pressures and thermal conduction to be dominant at high pressures. The observations above suggest a gas–solid reaction mechanism. However, it was found that the burning rates of high density Al/CuO pellets are uniform with the nitrogen pressure (Egorshev et al., 2013). This observation suggests a condensed phase reaction mechanism. At the nano scale, *in-situ* TEM images of the Al/CuO nanothermite reaction revealed a new condensed state mechanism, noted as reactive sintering (Sullivan et al., 2012). In this mechanism, reaction initiates at the Al/CuO interface, and the heat produced melts the adjacent products. The process is sustained due to capillary forces that deliver unreacted material to the reaction site. It was shown that reactive sintering occurs on a timescale of 10 μs, which is much faster than the burning time of ~1 ms. It was also shown that the nanostructure of the reactants is not preserved locally during the reaction. Similar TEM experiments showed that CuO releases oxygen faster than ignition (Egan et al., 2014). However, the condensed state reactions between Al and reduced CuO occurred on a timescale of 1 μs, which is much faster than the heterogeneous Al/oxygen reaction. Furthermore, SEM images illustrated large micron size spherical products after the Al/CuO reaction, and it was concluded that most of the reaction must have proceeded through a condensed phase mechanism (Jacob et al., 2015).

An exponential decrease of the flames speeds (from 100 to 1 m/s) with increasing the pellet density [20–70% of the theoretical maximum density (TMD)] was observed in the Al/MoO_3_ nanothermites (Sanders et al., 2007; Pantoya et al., 2009). This phenomenon was explained by a change in the combustion mechanisms from thermal conduction in low porosity pellets to mass convection in high porosity pellets. At high bulk densities, numerical modeling of the flame speed of Al/MoO_3_ based on conductive heat flow agrees with the experimental data (Kim, 2014). However, at low bulk densities, burn tube experiments in Asay et al. (2004) indicated that convection is the dominant transport mechanism, and the reduced particle size was elementary for enhancing convection (Prentice et al., 2006). Also, simple scaling arguments in Egan and Zachariah (2015) suggest that burning speeds on the order of 100 m/s can only be sustained by the transport of the condensed phases to the unreacted zone, since energy transfer via heat conduction and product gas convection/condensation is not enough to ignite the adjacent reactants.

The correlation between ignition and thermal analysis at low heating rates experiments illustrated four reaction steps in the Al/CuO combustion. It was suggested that the decomposition of CuO is the first step due to low activation energies, and that ignition is reached when 4% of the reaction enthalpy is released (Umbrajkar et al., 2006). Furthermore, ignitions of dense nanothermite on electrically heated filaments showed that ignition temperatures increase while pressurization rates decrease with the heating rate (Williams et al., 2013). The authors postulate that low temperature reactions prior to ignition alter the transport properties of the alumina layer and have important contribution on the ignition mechanism. In Zhou et al. (2017) and Yu et al. (2018), new methods to prepare core-shell Al/CuO thin film nanorods are described, and these nanothermites display several low-temperature reactions before the main exothermic reaction. These low temperature reactions are less resolved as the heating rate increases. Furthermore, the Al/CuO nanorods have the highest energy release at the Al rich conditions, and their onset temperature is independent of the stoichiometry. Other electrically heated filaments experiments showed that ignition delay is proportional to the oxide shell thickness, which supports the diffusion controlled mechanism of reaction (Chowdhury et al., 2010). The ignition temperature of Al/CuO was also correlated to the oxygen release temperature from CuO (Jian et al., 2013), but the activation energy for oxygen release decreased with the heating rate (Jian et al., 2014), which indicates that oxygen transport is limited.

The objective of this paper is to observe and analyze the ignition and burning speeds of Al/CuO nanothermites that are consolidated at different densities using physically mixed 40 nm nanopowders. The effect of nanoparticle ablation on the ignition delay and the effect of stoichiometry on the burning speeds are discussed. Similar experiments in literature focus mostly on the ignition delay and flame velocity. However, in this paper high speed and resolution images of the ignition and combustion processes illustrate specific macroscopic features of the Al/CuO mixtures prior and post ignition. A new method is proposed for calculating the speed of the burning front from the reacted zone to the unreacted zone. The heat and mass transfer processes that occur at the macroscopic scale determine the nanothermite performance both kinetically, in terms of the ignition delay and flame speeds, and thermodynamically, in terms of the combustion efficiency. It is found that the reactivity of the nanothermite mixture is strongly affected by the homogeneity of the reactants, and the fuel rich mixtures show reduced ignition delay, faster burning speeds, and higher pressurization rates compared to the stoichiometric mixtures.

## Experimental

### Materials and setup

Aluminum and copper oxide nano powders with APS (Aerodynamic Particle Size) diameters of 40 nm were purchased from US Research Nanomaterials Inc. The powders were added to a glass vial according to the stoichiometric or fuel rich ratios [equivalence ratio (ER) of 1.5 and 2], assuming that the aluminum nanoparticles are 65% active (based on the oxidation limits in a thermogravimetric analyzer). Hexane (10 mL) was added to the Al/CuO mixture (1,000 mg) and ultra-sonicated for 20 min to reduce agglomeration. The suspension was dried in a fume hood overnight on an evaporating plate, and then placed on a heating plate for 30 min to remove adsorbed species. The reactant mixture was consolidated at various densities using a pellet cast (6 mm diameter) and a hydraulic press, or packed lightly in an acrylic tube (7 mm inner diameter and 15 mm length). The final sample mass was 200 ± 20 mg.

**Figure 1:**
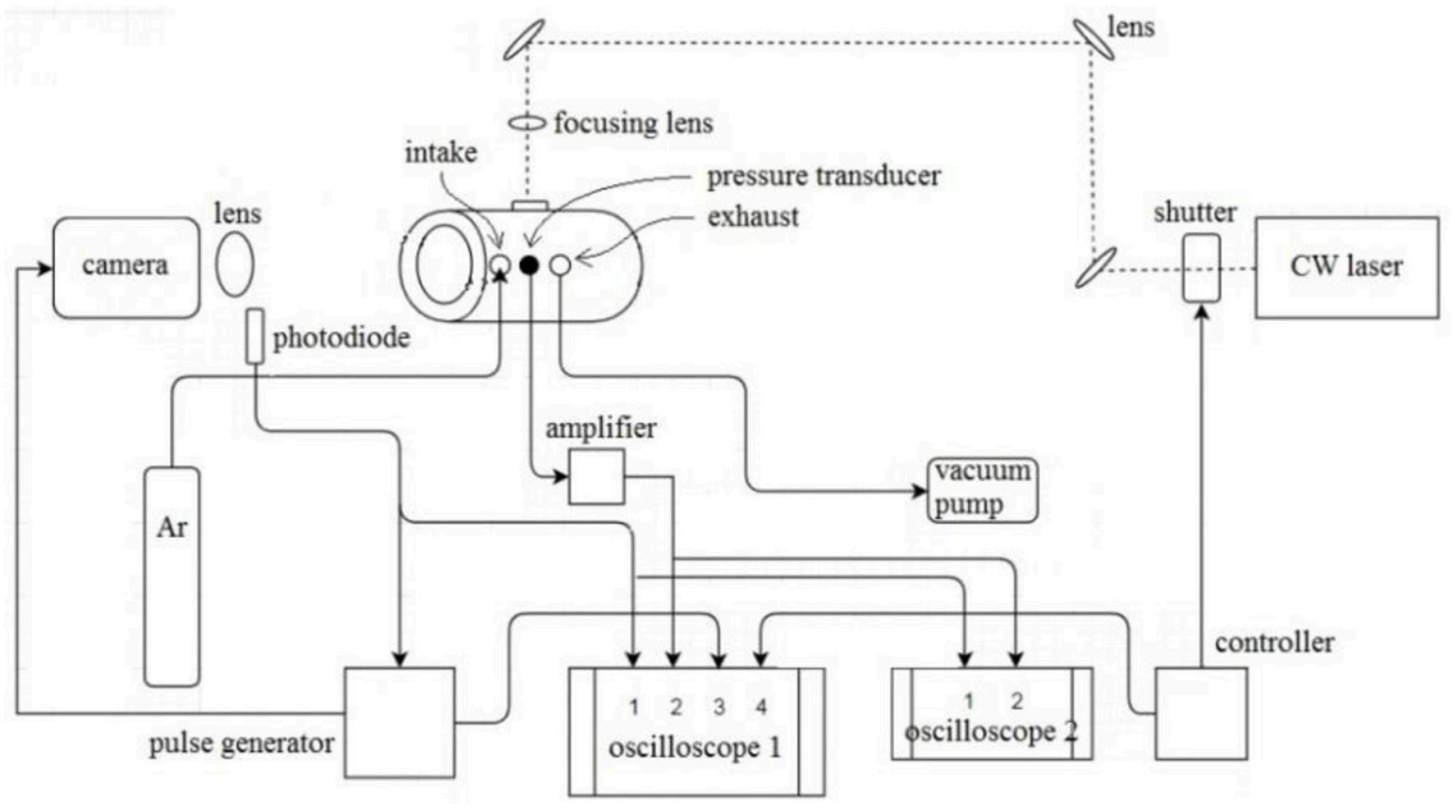
Diagram of the experimental setup for laser ignition and high speed imaging.

A diagram of the laser ignition and high speed imaging setup is shown in Figure 1. The pellet was held near the center of a cylindrical vessel (inner diameter of 100 mm), which was fitted with quartz windows on the ends. A continuous wave argon laser (3.5 W, 100 ms pulse duration) was used to ignite the pellet by heating its top surface, using a focusing lens to increase the power density from 225 W/cm^2^ to 40 kW/cm^2^. The signal from a photodiode (1 ns response time) was used to trigger a Phantom high speed camera, which was set to record at 200,000 fps and 500 ns exposure time with extreme dynamic range (EDR). A piezoelectric pressure transducer (1 μs response time) was installed on the vessel wall. Two oscilloscopes were used in order to capture high time resolution signals, and the full duration signals at lower time resolution. All tests were carried in argon at 1 atm.

### Methods

The ignition delay was measured using the high speed images, as the time difference between the initial laser light and the formation of the ignition front. This was also validated using the delay measured by the photodiode. The average burning speed within the nanothermite microstructure was estimated using the filtered high speed images, assuming a 2D planar burning front. Details of this procedure are described in section Average Burning Speed and Reaction Mechanism.

The normalized time-averaged photodiode and pressure signals were calculated according to

Y¯n=∫t1t2Ydt(t2−t1)m, where *Y* is the photodiode or pressure transducer signal, *t*_1_ and *t*_2_ are the initial and final times (according to full width at 20% maximum), and *m* is the sample mass.

Additionally, SEM (Scanning Electron Microscopy) images of the mixed Al/CuO reactants and the combustion products were taken, and EDX (Energy-dispersive X-ray spectroscopy) analysis of the products was performed.

## Results and discussion

### Reactant characterization

The as-received Al nanopowder is spherical, with an APS diameter and a specific surface area (SSA) of 40 nm and 30–50 m^2^/g, respectively. Furthermore, thermogravimetric analysis at a heating rate of 10°C/min up to 1,200°C indicates that the active Al content is 65% by mass. Under these conditions, the Al oxidizes fully to Al_2_O_3_. Theoretically, a 40 nm Al particle with an active content of 65% has a SSA of 53.33 m^2^/g. The higher SSA can be attributed to the particle size distribution and soft agglomeraton/aggregation of the nanoparticles. The as-received CuO nanopowder is nearly spherical, with an APS diameter and a SSA of 40 nm and 20 m^2^/g, respectively. Theoretically, a 40 nm CuO particle has a SSA of 23.44 m^2^/g. The SSA of the CuO nanopowder is much closer to the theoretical SSA compared to the Al nanopowder.

**Figure 2:**
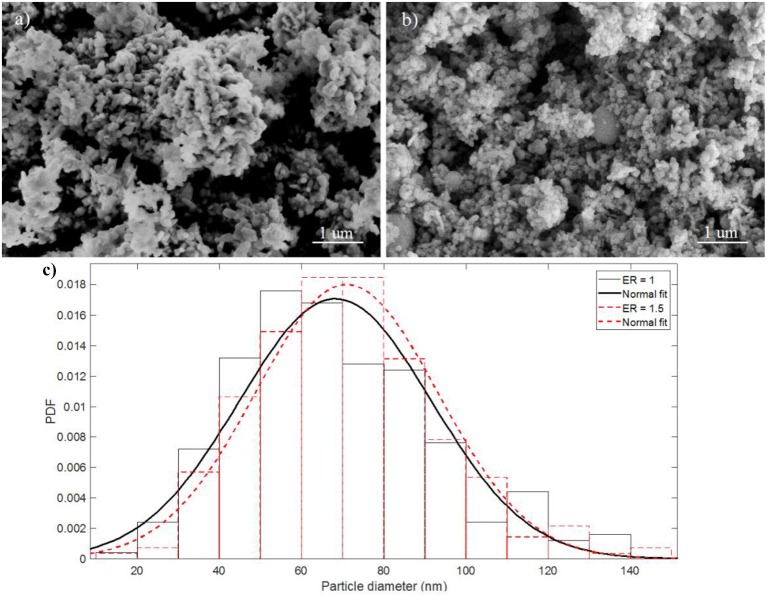
SEM images of as-prepared Al/CuO **(a)** stoichiometric mixture, ER = 1, and **(b)** Al rich mixture, ER = 1.5; **(c)** Particle size distribution of the stoichiometric and Al rich mixtures.

Figure 2 shows SEM images, at a magnifications of 50 k, of the as-prepared Al/CuO nanothemites with ER of 1 and 1.5, and their particle size distribution (based on the diameters of 300 particles at higher magnifications, 100 to 110 k). Refer to section Materials and Setup for the method of preparation. It can be seen that the stoichiometric mixture forms larger agglomerates, whereas the fuel rich mixture has larger particles. Since hexane is a nonpolar and hydrophobic liquid, the bulk density of the mixture may play a major role on the homogeneity of the suspension. The stoichiometric mixture has a higher bulk density compared to the fuel rich mixture due to the denser CuO. The most probable particle sizes are around 67 and 72 nm for the ER of 1 and 1.5, respectively. These values are larger than the APS diameters of the Al and CuO nanoparticles due to sintering during the sonication process.

The reference TMD for mixed Al/CuO nanopowders are calculated using the weight average of the Al, Al_2_O_3_, and CuO densities. The TMD for ERs of 1, 1.5, and 2 are 4.94, 4.61, and 4.37 g/cm^3^, respectively. The bulk density of the pellets and the lightly packed powders is relative to these TMD-values.

### High speed imaging of the ignition and flame propagation

**Figure 3:**
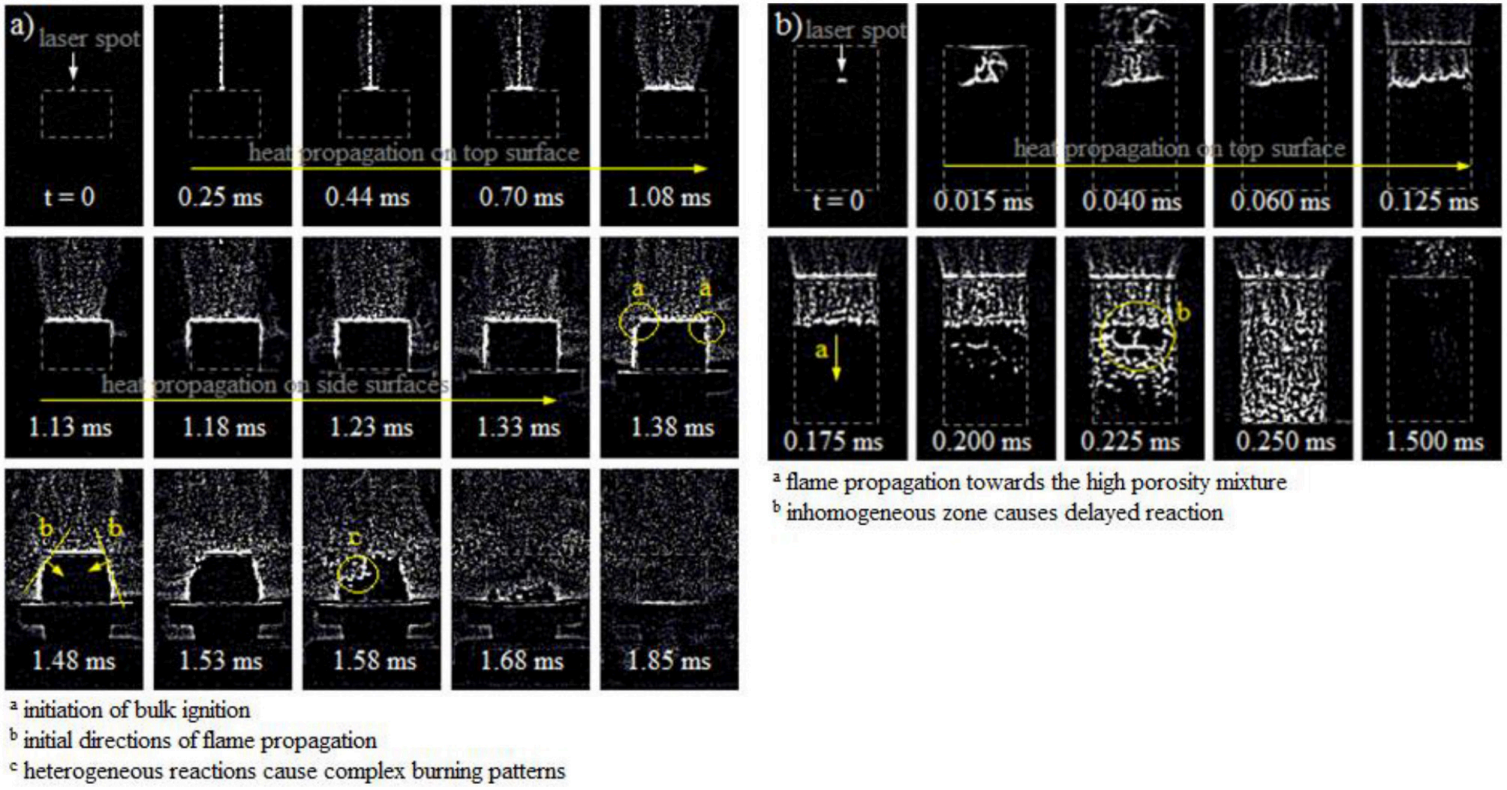
High speed frames of the ignition and propagation of the burn front within the Al/CuO mixtures for **(a)** pellet, and **(b)** lightly packed powder.

Figure 3 illustrates the high speed frames of the ignition and flame propagation in (a) an Al/CuO pellet with ER = 2 and density = 37.3 %TMD, and (b) Al/CuO powder in acrylic tube with ER = 2 and density = 13.8 %TMD. The 5 × 5 edge hipass filter in the Phantom PCC software is used to clearly show the location of the burning zones.

The camera frames in Figure 3a show that the laser creates significant nanopaticle ablation on the pellet surface. The heat produced by the laser propagates on the top and side surfaces due to the low thermal conductivities of the Al and CuO nanoparticles, while the nanoparticle ablation continues. The time frames indicate that heat propagates faster on the side surfaces, since the heat conductivity is enhanced axially due to the compaction. The bulk ignition occurs after 1.38 ms, around the top edge of the pellet. The edges shown in Figure 3a are preferential locations for ignition due to the elliptical shape of the laser beam. Then, the reaction self-propagates into the unburned mixture until the pellet is disintegrated at 1.85 ms. The flame front is well defined but non-planar due to the heterogeneous reaction sites. The low porosity of the pellet impedes the combustion gases to propagate into the unburned zone; hence the heat diffusion is a major component of the heat transfer that maintains the self-propagated sustainable reaction.

The photos in Figure 3b show that the laser also creates significant nanopaticle ablation on the surface of the lightly pressed powders. Similarly to the pellet, the heat propagates first on the top surface. The bulk ignition of the nanothermite powder starts after 0.175 ms, and the reaction propagates downwards into the unburned mixture. The reaction front is faster and disordered due to the high porous microstructure. The reaction zone reaches the end of the unburned mixture after 0.250 ms. It is expected that the large pores reduce the resistance to mass diffusion, and gaseous and molten products penetrate through the unburned zone to break the adjacent agglomerates and effectively reduce the diffusion lengths for the reaction; hence the mass convection is a major process in the self-propagated reaction.

### Ignition and ignition delay

The bulk ignition of the Al/CuO pellet was consistently initiated near the edges of the top surface, away from the laser heating spot. This phenomenon is caused by the combined effects of nanoparticle ablation and net heat transfer on the surface. Ignition temperature is not reached in the laser vicinity since the heated nanoparticles are continuously removed from the surface. Theoretically, the energy balance on the laser heated surface can be simplified to

$$\begin{array}{l} \rho c_{p}\frac{\partial T}{\partial t}=\alpha {\overset{\cdot }{Q}}_{laser}-\left({\overset{\cdot }{Q}}_{mix}+{\overset{\cdot }{Q}}_{Ar}\right)+{\overset{\cdot }{Q}}_{chem} \end{array}$$

here ρ is the packing density, *c*_*p*_ is the effective specific heat capacity, α is the absorption coefficient, ${\overset{\cdot }{Q}}_{laser}$ is the constant laser irradiance, ${\overset{\cdot }{Q}}_{mix}$ is the rate of heat transfer due to heat conduction and convection within the mixture, ${\overset{\cdot }{Q}}_{Ar}$ is the rate of heat convection and radiation to the argon, and ${\overset{\cdot }{Q}}_{chem}$ is the rate of heat release by the nanothermite reaction where its value depends on the local temperature relative to *T*_*ign*_, the ignition temperature

$$\begin{array}{l} {\overset{\cdot }{Q}}_{chem}=0\;for\;T<T_{ign}\\ {\overset{\cdot }{Q}}_{chem}>0\;for\;T>T_{ign} \end{array}$$

The ignition temperature is reached by minimizing the $\left({\overset{\cdot }{Q}}_{mix}+{\overset{\cdot }{Q}}_{Ar}\right)$ term. It can be assumed that the heat convection to the argon is much lower compared to the heat transfer within the mixture, which is dominated by the heat conduction since gas generation is very low before ignition. Hence the optimal conditions for the bulk ignition (thermal runaway) is around the edge of the laser heated surface, where the net heat transfer losses and nanoparticle ablation are minimized.

**Figure 4:**
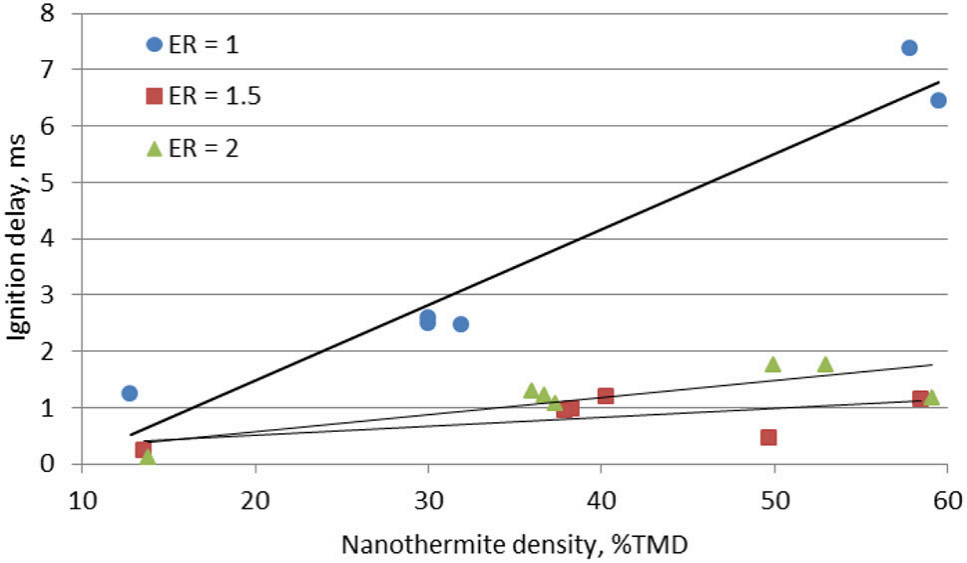
Ignition delays of the Al/CuO mixtures at different packing densities.

Figure 4 shows that the ignition delays increases with the packing density and the fuel rich mixtures have reduced ignition delays. As explained above, the ignition delay is controlled by the effective thermal conductivity coefficient (i.e., the porosity weighted average of the heat conductivities for the Al/CuO solid phase and the argon fluid phase). It has been shown that thermal conductivity increases linearly and absorption coefficient is uniform with density in consolidated aluminum pellets (40–75%TMD) (Stacy et al., 2014), and the same trend is expected to exist for Al/CuO. Therefore ignition is delayed in the denser pellets due to the larger heat diffusion on the surface and within the pellet. On the other hand, for a fixed density, the fuel rich mixture with ER = 1.5 has the shortest ignition delay. This suggests that the ignition delay is controlled by both the thermal properties of the mixture and the Al/CuO interfacial homogeneity, which controls the decomposition of CuO that provides the O_2_ required for the ignition. If the mixture is too fuel rich (ER = 2), the additional Al enhances the overall thermal conductivity, which delays the ignition.

It should be noted that the alumina passivation layer has a significant effect on the thermal properties of the aluminum nanoparticles used in these experiments. The thermal conductivities Al_2_O_3_ and CuO are equivalent, whereas the thermal conductivity of Al is an order of magnitude larger. Furthermore, the thermal conductivity of these components decreases with temperature (Kaye and Laby, 2018). The effective thermal conductivity is higher in the Al rich mixtures, and the laser heating propagates faster on the surface to reach the optimal conditions for ignition.

### Average burning speed and reaction mechanism

**Figure 5:**
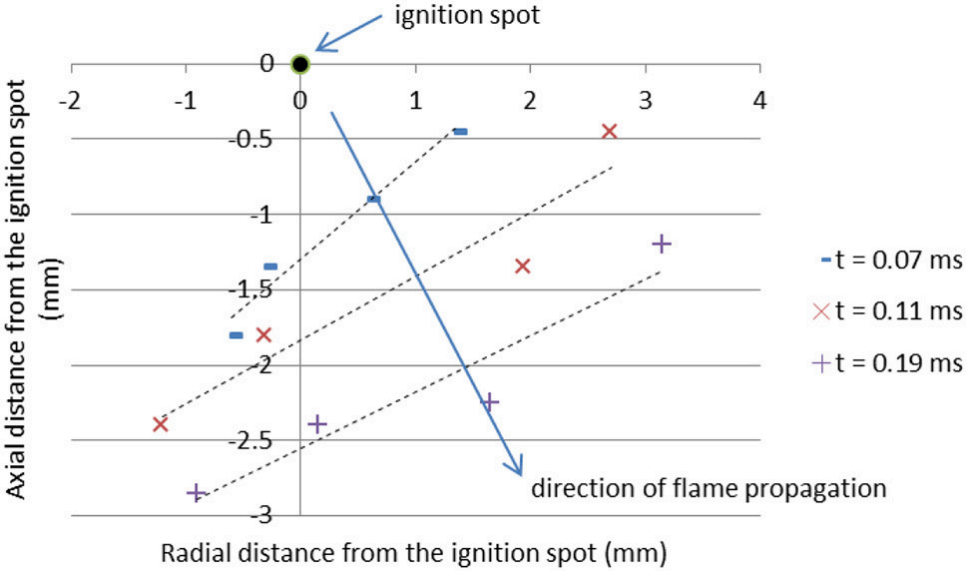
2D visualization of the flame propagation within an Al/CuO pellet.

In order to visualize the propagation of the burning front, the location of the bulk ignition is assigned to position (0, 0) and *t* = 0 on the 2D graph shown in Figure 5. Then, four points are selected on the flame front at different time frames and fitted with a line of best fit. The velocity of the flame front is calculated as

$$\begin{array}{l} v=\frac{d_{i}}{t_{i}} \end{array}$$

where *d*_*i*_ is the orthogonal distance, and *t*_*i*_ is the time from the ignition spot to the *i*^*th*^ frame. The average value of these flame speeds is assumed to be the average burning speed characteristic to the pellet. It should be noted that bulk ignition does not occur at a single location (refer to Figure 3a); in this paper, the flame speeds are calculated using the reference ignition spot that provides that fastest propagation. The combustion front of the nanothermite has complex 3D burning features; however, in this paper it is assumed that the fastest 2D flame speeds define a specific Al/CuO mixture.

**Figure 6:**
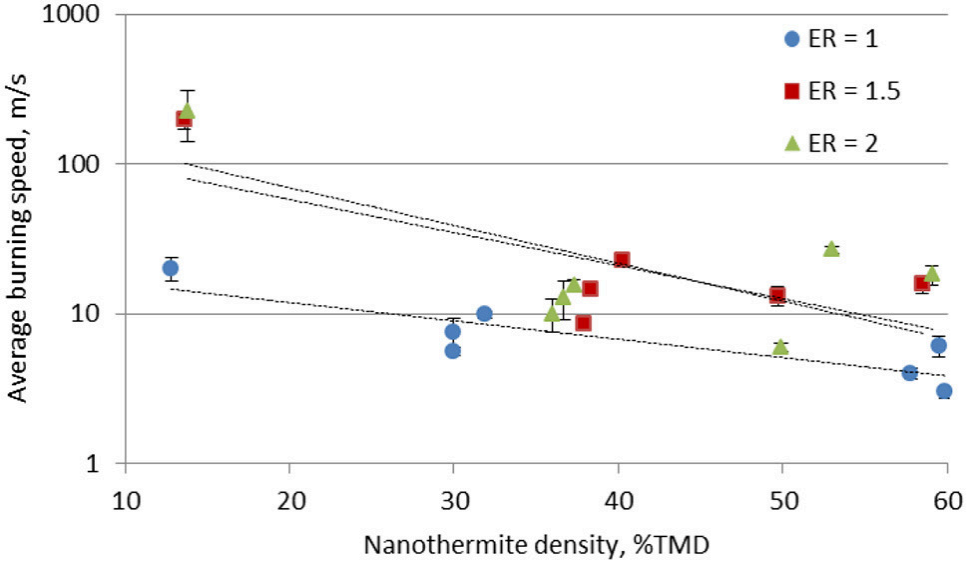
Average burning speeds in Al/CuO nanothermites at different packing densities.

Figure 6 shows the average burning speeds of the stoichiometric and the fuel rich Al/CuO mixtures. Two regimes are observed: the burning speed is on the order of 10 m/s in the consolidated pellets, and increases to the order of 100 m/s in the lightly pressed nanothermites. The tube confinement of the lightly pressed nanothermites plays a role on the flame speeds since the tube alters the rate of pressurization during combustion and the heat transfer to the argon; however, these effects are expected to be less significant than the porosity within the unreacted mixture.

A similar trend was observed in Al/WO_3_ nanothermites (Prentice et al., 2006), and 80 nm Al nanoparticles/CuO nanorods (Apperson et al., 2007). It should be noted that the burning speeds obtained in these experiments are relatively low compared to literature data (Apperson et al., 2007) since the 40 nm Al particles have significantly more alumina mass, which reduces the thermal conductivity. The 80 nm Al particles have an active content of 80% (Gromov and Teipel, 2014), compared to 65% of the Al particles used in these experiments. The effect of the large alumina shell mass is more pronounced in the consolidated pellets due to the higher local concentration of the alumina. The flame propagation is reduced since the pressed alumina shells increase the diffusion resistance.

To further examine the effects of the bulk density on the burning velocity, the Andreev number, *An*, is examined. This has been proposed recently to explain the transition from conductive to convective reactive flow in porous media (Weismiller et al., 2009). The *An* number is defined as the ratio of the convective heat transfer coefficient to the heat conduction coefficient,

$$\begin{array}{l} An=\frac{\rho ud_{\varepsilon }c_{p}}{k_{g}} \end{array}$$

where ρ is the packing density of the nanothermite, *u* is the average burning velocity, *d*_ε_ is the mean pore diameter, *c*_*p*_ is the specific heat capacity of the nanothermite, and *k*_*g*_ is the thermal conductivity of the gas. The mean pore diameter is found using Kozeny's equation for spherical particles (Skorokhod et al., 1988),

$$\begin{array}{l} d_{\varepsilon }=\frac{2}{3}\left(\frac{\varepsilon }{1-\varepsilon }\right)d_{p} \end{array}$$

where ε is the porosity, and *d*_*p*_ is the mean particle diameter. The specific heat under constant pressure is determined using the porosity averaged heat capacities of the solid and gas phases. The specific heat capacity and thermal conductivity values are examined at 2,000°K (Murphy and Arundell, 1994; NIST, 2017), assuming that the adiabatic flame temperature of 2,800°K is not reached. Also, it is assumed that the gas phase is a mixture of argon and oxygen, which is the major gaseous species during the reaction (Baijot et al., 2015).

Table 1 outlines the Andreev number for the stoichiometric and fuel rich Al/CuO mixtures. Generally, *An* is between 1 and 10 in the slow burning regime, indicating that heat conduction is significant for the self-sustaining reaction. In the fast burning regime, *An* is between 100 and 1,000, which indicates that thermal and mass convection drive the flame propagation.

**Table 1:** Andreev numbers for the Al/CuO mixtures at different packing densities.

**ER** = **1**							
%TMD	12.8	31.9	30	30	57.8	59.5	59.9
An	16.5	6.5	5.1	3.8	1.7	2.5	1.2
**ER** = **1.5**							
%TMD	13.6	37.9	38.3	40.3	58.5	49.7	
An	151.0	4.9	8.3	12.5	6.2	6.2	
**ER** = **2**							
%TMD	13.8	36.7	36	37.3	59.1	53	49.9
An	165.6	7.2	5.7	8.6	6.9	11.5	2.7

Overall, the fuel rich Al/CuO mixtures generated higher flame speeds than the stoichiometric mixtures. Similarly, maximum pressures and burn speeds in pressure cell experiments were obtained under fuel rich conditions (ER = 1.1) (Sanders et al., 2007), and this was assumed to be due to higher thermal conductivities (Jacob et al., 2017). Fuel rich Al/MO_3_ also showed improved propagation velocities compared to stoichiometric mixtures, due to optimum gas and liquid Mo generation that enhances the convective heat transfer (Son et al., 2007). As noted in Table 1, the fast burning regime is reached in the fuel rich lightly packed mixtures, but not in the stoichiometric mixture. These observations indicate that the mixture homogeneity is a critical parameter for fast flame propagation, and the local reaction rates control the gas generation that drives the convective burning. In Apperson et al. (2007), the self-assembled Al/CuO composites produced higher combustion rates compared to the physically mixed powders due to the larger interfacial area.

**Figure 7:**
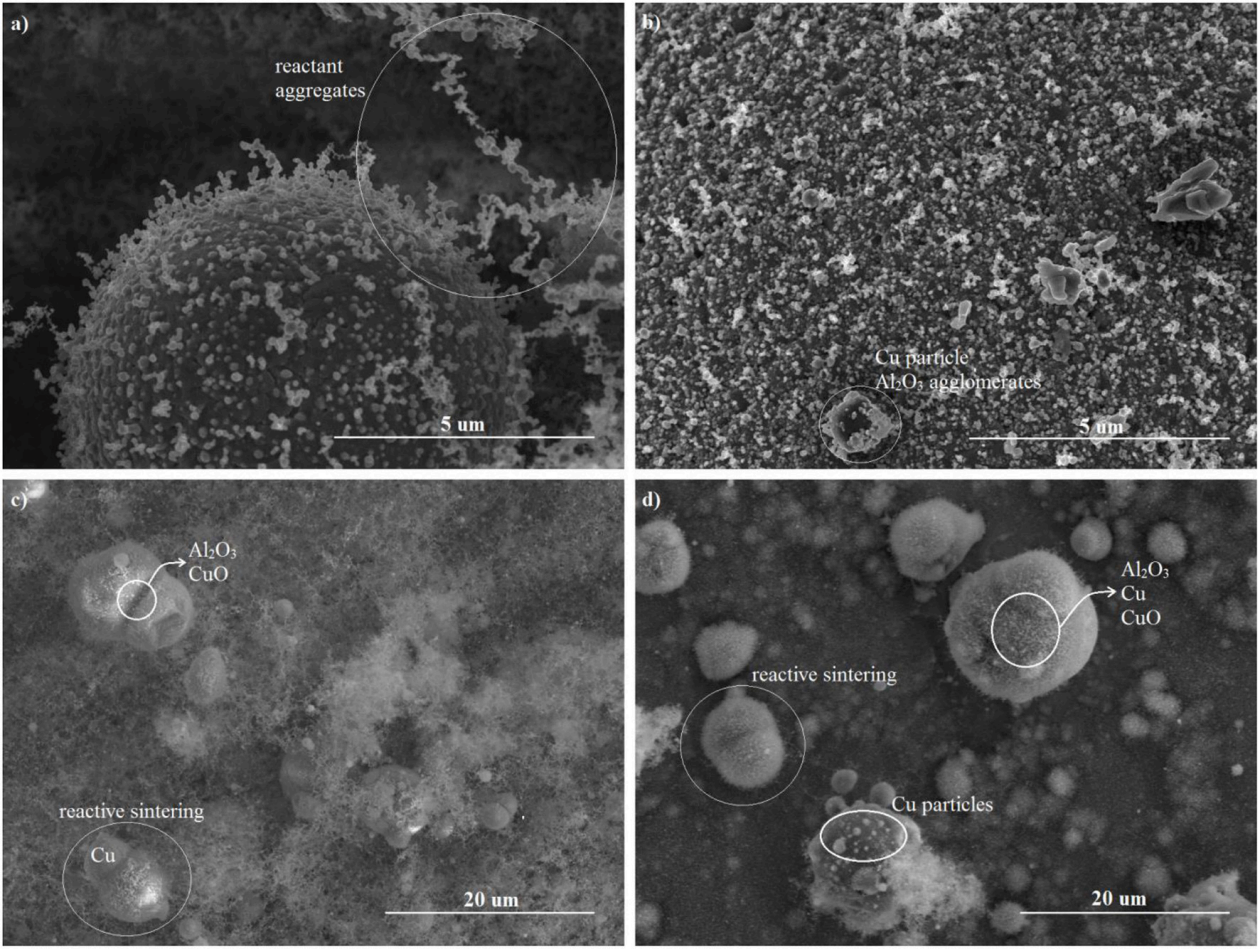
Post-combustion SEM of high density pellets with **(a)** ER = 1 (59.5 %TMD) and **(b)** ER = 1.5 (58.5 %TMD); and low density powders with **(c)** ER = 1 (12.8 %TMD) and **(d)** ER = 1.5 (13.6 %TMD).

To further study the nanothermite reaction, post-combustion SEM images of the consolidated pellets and lightly packed powder under stoichiometric and fuel rich conditions are shown in Figure 7. Generally, the products of the nano scale reactants are on the micro scale, and composed of spherical Cu particles and aggregates of Al_2_O_3_ and Al_x_Cu_y_O_z_ intermetallics. Similar structures have been observed in (Jacob et al., 2015) and (Jacob et al., 2017).

Although the initial pellets densities of the stoichiometric and fuel rich mixture in Figures 7a,b are similar, the morphologies of the products are different. The reactant aggregates observed in the post-combustion of the stoichiometric pellet indicates a highly incomplete combustion. This is attributed to the larger reactant agglomerates, and inhomogeneous mixing, shown previously in Figure 2a as compared to Figure 2b. The products of the lightly pressed nanothermites in Figures 7c,d show that reactive sintering occurs in the highly porous mixtures. Despite the high porosity that promotes convection, the condensed phase reactions are much faster than the heterogeneous reactions. For example, the reaction time of Al and CuO in condensed phase is on the order of 1 μs (Egan et al., 2014), much faster than the burning time of Al nanoparticles in an oxygen atmosphere which is on the order of 100 μs (Bazyn et al., 2006). It should be noted that significant reactant aggregates exist in the products of the stoichiometric loose powders (Figure 7c), which is evidence of an incomplete combustion. Incomplete Al/CuO reactions have also been reported in Jacob et al. (2017) and Glavier et al. (2015). The large ignition delay and the low burning speeds of the stoichiometric mixtures are mainly caused by inhomogeneous mixing of the reactants that limits the local Al-CuO reactions in the condensed and heterogeneous phase. Consequently, this limits the gas generation that promotes the mass convection, and the combustion temperature that promotes the heat conduction.

### Reaction performance of the pellets

The photodiode and pressure signals were analyzed for the different pellet densities in order to assess their performance. The key factors depend on the application. For example, propulsion and igniter applications required fast pressurization rates, whereas welding applications require high reaction temperatures. It should be noted that the pressure transducer was not installed in the reaction zone; hence the pressure signals are only used for comparison purpose.

**Figure 8:**
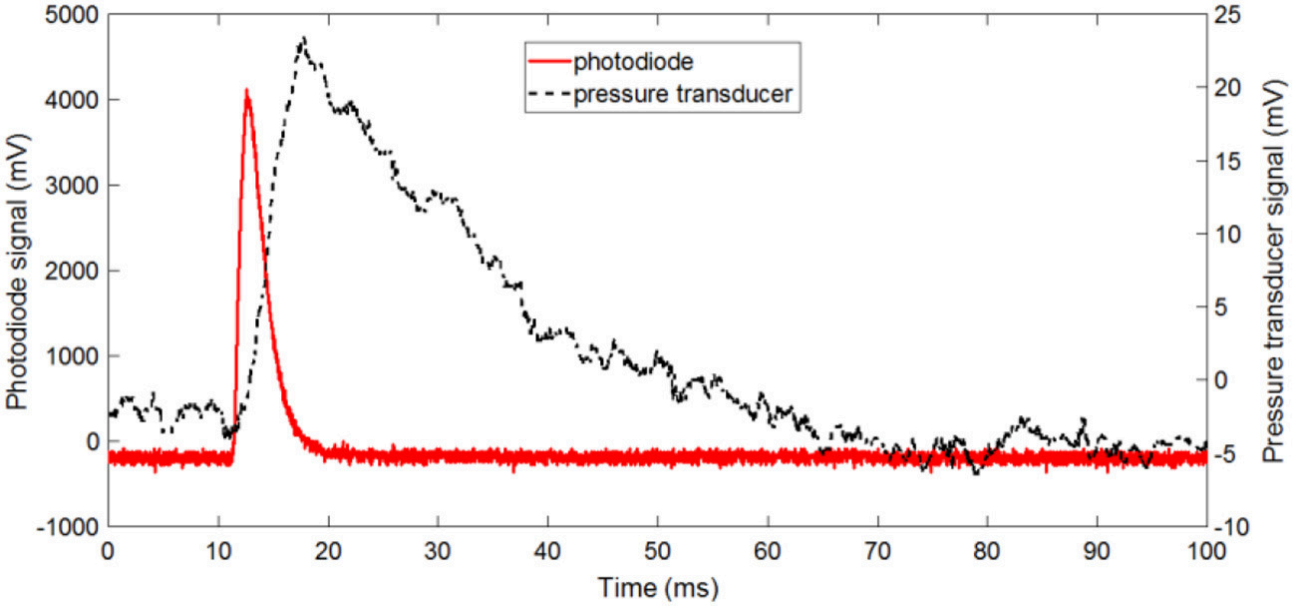
Raw photodiode and pressure signals for a high density pellet with ER = 1.5.

**Figure 9:**
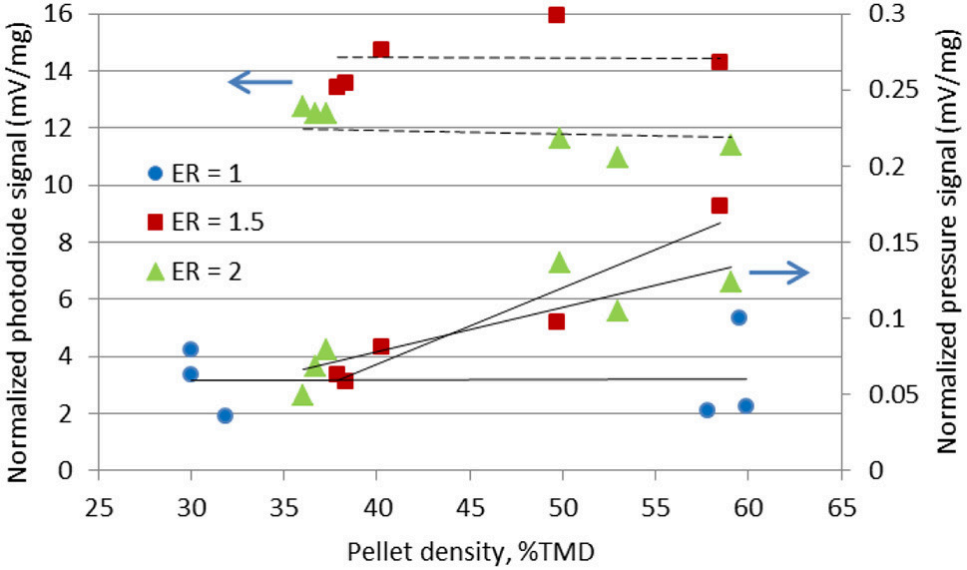
Normalized photodiode and pressure signals of the Al/CuO pellets at different packing density.

Figure 8 shows the raw signals of the photodiode and pressure transducer during the combustion of a high density Al/CuO pellet with ER = 1.5. The reaction occurs before the gas release, and the burn duration is faster than the pressurization time. It should be noted that some researchers observed the decomposition of CuO prior to the ignition; however, the pressure transducer in these experiments can only measure the overall pressurization near the vessel walls. Figure 9 shows the normalized and time-averaged photodiode and pressure signals of the Al/CuO pellets. The normalized time-averaged photodiode signal term represents the specific energy release rate by the reaction. This term is independent of the density, which indicates that the degree of oxidation of Al is similar. The mixtures with ER of 1.5 have a larger normalized photodiode signal compared to the mixture with ER of 2 since the extra Al in the richer mixture acts as a heat sink. The normalized time-averaged pressure signal increases linearly with the pellet density. A similar linear increase with the density was also observed for the pressurization rate, which is estimated from the slope of the raw pressure curve. The higher pressure rates in the denser pellets are caused by the reduced volume for gas expansion, which is also predicted by a theoretical model assuming local thermodynamic equilibrium (Baijot et al., 2015). Furthermore, it is expected that the consolidated Al and CuO reactants have more reactive interfaces, which enhance the initial reaction rates in the condensed phase. In Glavier et al. (2015), the maximum pressure and pressurization rates of Al/CuO pellets also increase with the %TMD. Theoretically, much higher pressures are predicted since the actual gas phase chemistry is unknown. It should be noted that the normalized pressure signals of the stoichiometric Al/CuO pellets are low compared to the fuel rich mixtures, and independent of density. This is further indication that the local reaction rates are inhibited by the homogeneity of the mixture.

The burning durations of the Al/CuO nanothermites in these experiments, as measured by the duration of the photodiode signal, are an order of magnitude longer than the durations of flame propagation. This indicates that the degree of oxidation of the Al nanoparticles is limited by the heterogeneous Al–O_2_ reactions. In Jacob et al. (2017), temporal temperature measurements in Al/CuO nanopowders were near the flame temperature of Al particles in air (~4,000°K). Thus, in order to improve the reactivity of these nanothermites, the Al-CuO interfaces should be optimized to ensure that most of the reaction initiates in the condensed phase, and oxygen decomposition from the CuO can readily react with the Al.

## Conclusions

The experiments show that the reactivity of physically mixed Al/CuO nanothermites is highly sensitive to the homogeneity, ER and packing density of the mixture. The fuel rich mixtures burned much faster than stoichiometric mixtures due to formation of smaller agglomerates in the reactants. The ignition delay on the pellet surface is controlled by the nanoparticle ablation and the net heat transfer on the surface, such that the pellet edges are the preferential spots for the bulk ignition. The propagation speed of the burning front increases from an order of 10 m/s in the consolidated pellets (40–60%TMD) to an order of 100 m/s the in lightly packed powders (10%TMD). Enhanced flame speed is caused by a change in the controlling mechanism from heat conduction to mass convection with decreasing the packing density. The reactivity of the Al/CuO pellets increases generally with the packing density, whereas the normalized pressurization rate increases linearly with the density.

## Data availability

The raw data supporting the conclusions of this manuscript will be made available by the authors, without undue reservation, to any qualified researcher.

## Author contributions

FS obtained the experimental results, analyzed the data, and wrote the manuscript. MI and NC set up the experiments and provided the lab facilities. JW revised the manuscript and supported this research.

### Conflict of interest statement

The authors declare that the research was conducted in the absence of any commercial or financial relationships that could be construed as a potential conflict of interest.
